# Who Carries the Responsibility for Health Care Carbon Reduction?

**DOI:** 10.1002/hast.5008

**Published:** 2025-06-25

**Authors:** Cristina Richie, Gabrielle Samuel

**Keywords:** sustainable health care, decarbonization, policy, environmental bioethics, net‐zero emissions, sustainability

## Abstract

*Policy‐makers, doctors, organizations, and academics who are persuaded that health care decarbonization is an ethical mandate are grappling with ethical and effective implementation of measures to support this goal. Health care carbon‐mitigation strategies (both proposed and potential) at individual, regional (city, state, or council), national, and international levels have already been analyzed to different degrees; however, a comparative analysis of strategies at each of these levels that takes into account bioethical issues such as autonomy, responsibility, and shared decision‐making has not previously been conducted (though some analysis between national and international efforts has occurred). This essay offers a comparative analysis of the ethical aspects of health care carbon reduction across these levels, including considerations of responsibility and of the potential for efforts at each level to actually impact climate change*.

## Essay

Health care, understood as a sector of the overall economy, should have an ethical interest in reducing its contribution to climate change. Health care contributes 4 to 5 percent of the total global greenhouse gas emissions,^1^ including carbon dioxide (herein, “carbon”), and climate affects human health through climate‐related health hazards. In fact, the World Health Organization estimates that one out of four deaths globally is caused by environmental factors, with over four million deaths annually due to air pollution alone.^2^ Many millions more people have illnesses caused by climate‐related health hazards. Since it is the health care sector that responds to these arguably preventable illnesses and diseases, this sector ought to reduce its own emissions. It should do so, first, simply to reduce climate change as part of broader environmental ethics and, second, to contribute to reductions in the disease burden created by climate hazards, thereby supporting public health ethics.

While the moral requirement for health care to reduce its emissions is generally uncontested by ethicists,^3^ many health care organizations and stakeholders—such as patients, physicians, and administrators—maintain a medical exceptionalism about the climate crisis, arguing that medicine should be excluded from requirements to reduce carbon because “patients’ health could be jeopardised by climate change mitigation.”^4^ Resistance to the idea that the health care sector has a role to take in carbon mitigation is suggested by the sector's lack of engagement with signifiers that proactively endorse environmental awareness. For instance, as of March 2022, 55 percent (61/111) of U.S. medical organizations’ public‐facing websites had no evidence of reference to climate change.^5^ Sustainability can occur only if it is recognized as an issue within the scope of health care.

Given the disjuncture between attitudes toward climate change as a medical issue and toward obligations to decarbonize health care, current debates in biomedical ethics are twofold. The first debate is about whether health care has an obligation to reduce carbon; we accept that it does. The second debate is about how carbon reduction should happen. Policy‐makers, doctors, organizations, and academics who are persuaded that health care decarbonization is an ethical mandate are grappling with ethical and effective implementation. Health care carbon‐mitigation strategies (both proposed and potential) can be envisioned at individual,^6^ regional (city, state, or council),^7^ national,^8^ and international levels.^9^ While these levels have been studied to different degrees, a comparative analysis of strategies at each of these levels, taking into account bioethical issues such as autonomy, responsibility, and shared decision‐making, has not been adequately conducted. (There has been some comparative analysis of national and international mitigation efforts.^10^)

The broader ethics scholarship stresses that environmental harms should be addressed through collective responsibilities, and in a way that assigns responsibilities proportionally to ability.^11^ This essay seeks to make clearer what the collective responsibilities are for health care carbon reduction, by undertaking a comparative analysis of the ethical issues of health care carbon reduction across levels from the individual to the international; this shall include considering, level by level, the responsibilities and the potential to actually impact climate change. (For a summary of actions that stakeholders can take at these different levels to reduce health care carbon emissions, see table 1.) While the analysis of potential is somewhat speculative, we offer it as a heuristic to explore, rather than an argument from quantitative data on carbon footprints in health care—which are neither comprehensive nor consistent between countries. We will bracket the discussion on climate‐adaptation strategies for health care, which have been proposed elsewhere^12^ and are outside of the scope of this paper.

**Table 1:** Health Care Emission‐Reduction Summary

Level of action	Stakeholder	Examples
**Patient or consumer**	Patient Clinician	Carbon budget Green consent
**Regional**	Local individuals, groups of individuals, or communities of practice who work in health care; health care institutions	Advocacy Code of conduct for best practice Green funding schemes
**National**	National regulators National health systems State government Federal government	Voluntary guidance Mandatory guidelines Binding legislation
**International**	World Health Organization and United Nations UN Conference of the Parties Health(‐related) agencies or protocols	Nonbinding or binding legislation

## Carbon Emission Reduction in Individual Health Care

In the United Kingdom, to consider one example, individual or household carbon footprints account for 26 percent of national greenhouse gas emissions.^13^ Not included in this figure is the additional 640 kilograms of health care carbon per capita.^14^ Action at the individual level can make health care more sustainable. One approach is to give responsibility to individual patients for their health care emissions. This can be achieved by educating patients about health care carbon impact and then empowering them to decide if, and when, to use medical treatments.

For instance, some doctors have rejected the blanket recommendation for an annual checkup as unnecessary, instead advocating for checkups based on individual medical profiles.^15^ If a person knew the emissions associated with a doctor's appointment—in the United Kingdom, an estimated 50 kilograms of carbon for an outpatient appointment, based on data for primary and secondary care as well as energy used by the building^16^—then they might choose to forgo other outpatient appointments like so‐called wellness checkups or to have them less frequently, perhaps every other year. This would be a voluntary choice that would, moreover, be based on patient autonomy in the right to refuse treatment.^17^

Alternatively, a patient might also ask for lower‐emission alternatives in clinically indicated medicine.^18^ *Green informed consent*, defined as “the sharing of climate information with patients, offering options for lower‐carbon health care, and accepting the patient's right to decline treatments which are deemed too carbon intensive for their values,” allows patients to receive information on the resource impact of treatments, and make a decision with this factor in mind.^19^

***Benefits of individual‐level emission reduction***. Placing some of the focus of health care emission minimization at the individual level gives people the opportunity to get involved, acting as an invitation to participate in climate change minimization. Reduction of individual level emissions can have at least two benefits. First, facilitating green health care choices might have a positive mental health impact on a person by giving that person agency to take responsibility for their emissions. Climate participation and climate activism in health care^20^ meet with clinical ethics at the point of the shared decision‐making process. A 2024 Dutch national survey of 9,371 patients found that 69 percent considered environmental sustainability an important aspect of health care delivery, and 73 percent expressed a willingness to incorporate sustainability factors into their health care decisions.^21^ Studies from the United States,^22^ Germany,^23^ and the Netherlands^24^ report similar trends in patients’ desires for more sustainable health care choices.

Second, individual efforts to reduce health care emissions could occur by avoiding unnecessary treatments—either through making efforts to maintain one's health—and thereby not needing medical attention—or through being proactive when medical options are offered. Patients can ask if the treatments being offered are clinically necessary or if there are nonmedical alternatives. It might be surprising that clinically unnecessary treatments are offered to patients, but given the trends in medicalization^25^ and medical overuse and overprescribing, not everything that is offered under the guise of health care is really that. Patient autonomy ensures that individuals can refuse or resist unnecessary treatments that are documented sources of unnecessary carbon emissions,^26^ as well as of potential health harms. To this end, it should be noted that individual‐level carbon reduction, if implemented based on informed decision‐making through green consent, may have the cobenefits of reducing risks of medical error, medical death, and harmful side effects of medication.

***Drawbacks of individual‐level emission reduction***. Addressing carbon reduction in health care at the individual level poses a number of challenges. It may be seen as ethically problematic to ask, expect, or encourage individual emission minimization due to the vulnerabilities of being ill. Feelings of guilt for incurring a health condition or stigma from certain types of diseases could be problematically compounded if tied to negative carbon impacts. Placing responsibility for emission savings on patients might also increase the informational burden on people who already need to digest a wealth of health‐related information and may increase any ecological despair patient might feel.^27^ It also reproduces the neoliberal agenda of offloading responsibility from institutions to individuals, distracting from the need for broader sociopolitical change.^28^ From a shared decision‐making perspective, too, placing this responsibility on the patient is problematic, as it is too demanding of the patient. Other ethical externalities might include the perpetuation of structural injustices. For example, due to gender expectations, women more often than men feel pressure to be sacrificial,^29^ so women may be disproportionately harmed if they feel social pressure to forgo medicine^30^ due to carbon impact.

As with other environmental choices, the ethics of indirectly nudging people toward sustainability (for instance, through carbon taxes on luxury medical goods) and removing the option to use resources that are environmentally harmful (for instance, banning certain types of anesthetic gases^31^) remain contested.^32^ Practically, individual health care emission mitigation, dependent on the voluntary choices of patients, also simply might not make a significant difference with the problem of climate change.

## Carbon Emission Reduction in Regional Health Care

Health care regions may be conscribed by city, state, or council, depending on the country. Health care carbon reduction at the regional level may include, on the one hand, bottom‐up local advocacy or initiatives accepting ethical responsibilities to promote changes in local health care practices and, on the other hand, top‐down enforcement from health care facilities, which take on responsibilities in the form of low‐emission policies. Possible regional strategies include local hospitals’ switching to renewable energy sources or generating their own renewable energy,^33^ driving a net‐zero agenda through mandatory or voluntary “best practice” guidance (concerning, for example, transportation to work,^34^ the recycling of wastewater, and reducing plastic use^35^), low‐emission care‐pathway alternatives^36^ (such as the use of virtual care^37^), and mandatory implementation of procurement, waste, or other policies that take emissions into account. Bottom‐up initiatives can also include local community‐level action to support community health, which could ultimately reduce the need for health care. Examples could include creating more green space in a health care facility or in the local community and supporting community campaigns to reduce air pollution.^38^

***Benefits of regional‐level emission‐reduction efforts***. Regional efforts give those health care providers and facilities who want to reduce their emissions an opportunity to accept ethical responsibility and change their own practices. Such efforts can lead to quick changes in practice without the requirement for formal policies or broader legislation. These efforts can be context specific, be relevant to local communities, address clinical and community needs, and draw on local expertise in departments and facilities. Such context specificity is key to ensuring best ethics practice and to ensuring that local voices are heard and considered during the implementation of climate change‐mitigation strategies.

Furthermore, local efforts are more likely to be agile and transferable and can be more easily tailored to different circumstances and needs. Success at the local level can be inspiring for other local communities, and communities can contribute to a cross‐pollination of knowledge. Local initiatives can also be scaled: one example is the international planetary health report card, which was first developed in 2019 by a group of University of California medical students as an institutional advocacy tool. Finally, regional efforts may take the pressure off the individual patient to minimize health care carbon.

***Drawbacks of regional‐level emission‐reduction efforts***. Because of their context specificity, regional efforts, while being agile and transferable and sometimes scalable, are nonetheless often difficult to expand to other regions given differences in health care settings. Difference between the communities and populations served may mean that changes in one region are not relevant to another. For instance, initiatives advocating for public and cycle transport are likely to have more effect for city‐based health care facilities than for rural settings. Lessons learned may remain at the local level, making it hard to share knowledge, scale up, and achieve a meaningful overall impact. Local initiatives may also be seen as less important than national health care initiatives because they are implemented bottom up and may have less impact on overall health care emissions. Furthermore, while regional efforts align with the common conception that thinking globally means acting locally,^39^ the lack of scalability means that such initiatives may not be sustainable. Health care facilities are often complex systems with many moving parts, making change difficult. Precedent suggests that obtaining buy‐in for local‐led and bottom‐up initiatives is extremely difficult. This is especially true if such changes require initial financial investment because health care facilities may perceive the changes to require adjustments to other priorities when resources are limited. This means that local initiatives are unlikely to have long‐lasting legacies, and effort, resources, and time may therefore be wasted.

Another consideration is that, if local initiatives in the clinic are voluntary, rather than organizationally supported, then creating sustained behavior change may be difficult. Voluntary initiatives place greater burdens on the workforce: many health care professionals lack the resources, time, or energy to advocate for or implement environmental changes over the long term.^40^ Finally, initiatives that rely on voluntary contributions raise questions of value—in terms of the workers themselves (who do not receive remuneration for their efforts) and the work they are doing (which there is a lack of incentives to conduct, with an implicit assumption that volunteers are doing it, so institutions do not have to).

## Carbon Emission Reduction in National Health Care

Placing the ethical responsibility for health care carbon reduction at the national level makes sense because countries have the ability to implement such changes. Such responsibility can take the form of legally binding carbon‐minimization measures. In the United Kingdom, the Climate Change Act of 2008 led the National Health Service to adopt the Carbon Reduction Strategy for England.^41^ Scotland has its own NHS Climate Change Plan,^42^ and the NHS has recently set a more aggressive carbon‐reduction scheme and is aiming for a “net‐zero” goal.^43^ However, national health care strategies may also be more reliant on voluntary participation, with governments sharing responsibility with those who sign up for inclusion. One example is the Dutch government's Green Deal on Sustainable Healthcare, which has gathered over two hundred participants from the health care sector, including “healthcare providers, pharmaceutical companies, and a range of profit and non‐profit organisations”^44^ as well as insurers and the Netherlands’ Ministry of Health, Welfare and Sport. The program operates in conjunction with the overarching Dutch Green deal. Other voluntary models include institutional initiatives that link affiliate hospitals to carbon‐reduction commitments, such as Practice GreenHealth, which self‐describes as a “sustainable health care organization, delivering environmental solutions to more than 1,500 hospitals and health systems in the United States and Canada”;^45^ the now‐dissolved Healthier Hospitals Initiative, a United States‐based campaign to implement sustainable measures in the health care sector, focusing on healthier food, leaner energy, less waste, safer chemicals, and engaged leadership;^46^ and the Catholic Health Association, which has a focus on the environment and is “the largest group of nonprofit health care providers in the [United States],” comprising “more than 650 hospitals and 1,600 long‐term care and other health facilities in all 50 states.”^47^

***Benefits of emission reduction at the national level***. If governments orchestrate policies, then there will be more oversight to ensure that the policies are followed. And while there might be policy gaps in national carbon‐minimization efforts, as long as some policies or targets are disseminated, accountability is possible. If health care providers choose their own targets, they will be beholden to stakeholders and the public

Carbon saving schemes like “net zero” can cover the entire health care system, ensuring that every sector is participating in carbon reduction. Thus, national oversight of health care carbon can lead to much greater impact in sustainability through the coherence of carbon‐reduction plans. This is an important ethical aspect of justice in resource allocation. Although higher carbon sectors (like surgery) may have more of an obligation to reduce carbon‐intensive practices to support impactful change, uniformity across regions will demonstrate solidarity.

National health care carbon reduction can also demonstrate solidarity with other international sustainability efforts. In many, but not all, countries, there is a governmental push for more corporate social responsibility. Taking on the ethical responsibility to reduce health care carbon at the national level demonstrates leadership in addressing a global, multisector problem. Climate change health hazards affect not only residents, but also people in other countries. As a matter of business ethics, all economic sectors should take steps to conserve natural resources, but given the organizational mission of health care, the health care sector ought to be at the fore of an effort aimed at safeguarding the health of all.

The U.K. model of national health care carbon reduction can and does cross borders and has influenced other countries, such as Norway,^48^ while also making environmental inroads in the medical supply chains. With this example to learn from, other nations have less excuse to avoid implementing a similar health care carbon‐reduction strategy.

***Drawbacks of emission reduction at the national level***. The narrative around nationally driven carbon‐reduction measures—whether legally binding or voluntary—may be misleading. For instance, the NHS “net zero” strategy, while commendable, has considerable environmental gaps. First, something that produces net‐zero emissions is not producing zero emissions: net‐zero health care still pollutes but offsets carbon to achieve net‐zero status. Second, carbon emissions are only one aspect of the environmental and climate issues facing society.^49^ Resource use, loss of biodiversity, ecosystem health, and climate change health hazards are not encompassed in the metric of carbon. In light of these considerations, “net zero” and similar phrases may be seen as an unethical form of “greenwashing.”

National health care carbon reduction also potentially undermines the sense of responsibility at other levels. If health care consumers and doctors feel that someone else is taking care of the climate problem, they may themselves take a backseat approach to sustainability.^50^

In terms of impact, if nations rely on voluntary measures for health care carbon reduction, then some of the largest contributors may opt out, while other, less impactful sectors become sustainable. Because ethical responsibility demands swift and immediate climate action, this approach may therefore not be enough. At the same time, legally binding national polices may be unattainable. Necessary supports—from administrations to clinician education to agreements with health care suppliers—all need to come together for real change to happen.

## Internationally Negotiated Carbon Emissions Reduction in Health Care

As a global issue, climate change transcends national boundaries. Emissions need to be reduced across all nations and jurisdictions, particularly in higher‐income countries that contribute more to emission levels and have more resources to invest in mitigation strategies. International coordination and cooperation to reduce global emissions takes place during the annual United Nations Conference of the Parties (COPs). During COP21 (in 2015), countries signed the Paris Agreement, a pledge to reduce emissions enough that global temperatures will not increase more than two degrees Celsius above preindustrial levels and to pursue efforts to limit that increase to 1.5 degrees Celsius above preindustrial levels.^51^ At the most recent conference, COP28, the international community committed to “transitioning away from fossil fuels in energy systems, in a just, orderly and equitable manner, accelerating action in this critical decade, so as to achieve net zero by 2050 in keeping with the science.”^52^

Commitments have also been made at the international level within the health sector: the Alliance for Transformative Action on Climate, a group of 80 countries, has pledged at the Minister of Health level to strengthen the climate resilience or reduce the greenhouse gas emissions of their health systems.^53^ During COP28's designated “health day,” 143 countries endorsed a declaration that aims to promote “steps to curb emissions and reduce waste in the health sector, such as by assessing the greenhouse gas emissions of health systems, and developing action plans, nationally determined decarbonization targets, and procurement standards for national health systems, including supply chains.”^54^ The World Health Organization's *Operational Framework for Building Climate Resilient and Low Carbon Health Systems*, released in November 2023, provides international guidance to achieve these emissions reductions.^55^

Other stakeholders and actors—including agencies such as the Pan American Health Organisation^56^ and the World Bank,^57^ as well as international networks and alliances^58^—including Health Care Without Harm^59^—are also working to ethically reduce health care emissions in the international arena. All of these offer a clear rationale and strategy for health care organizations to participate in pursuing or upholding global standards of climate action.

***Benefits of emission reduction at the international level***. Placing health care emissions reduction within this wider international context makes it more likely that health care reductions are part of a bigger push to address global emissions. In fact, if international agreements are followed, they have the potential to reduce global emissions by addressing the actions of the biggest emitters of greenhouse gases and driving the biggest changes in terms of investment in renewables.

Furthermore, such international agreements remove the possible burden on national health care to make a commitment to carbon reduction by making that commitment a predetermined factor in health care decisions. Within the parameters of national carbon reduction, the priority of health is maintained with the understanding that health care will be allocated, as other goods and services will be, in a way that is clear and supports the interests of environmental sustainability.^60^ Though international agreements are often seen as merely aspirational, they can make individual countries accountable to the rest of the world, requiring countries to enact their climate responsibilities. Consensus‐based COP activity contributes to international legal obligations by thickening the obligations of the parties to the underlying treaty. In fact, many lawsuits are currently seeking to hold nations and corporations accountable for their climate pledges through national legal systems.^61^

***Drawbacks of emission reduction at the international level***. Most COP agreements are not legally binding; they are aspirational accords between nations, signed by legal representatives. While some lawsuits are seeking to hold signing nations accountable—as in the 2020 example in which six young people from Portugal filed a climate action case against the European Union—the accords do not necessarily need to be put into practice—nations can ignore their responsibilities. Furthermore, even when countries try to implement these ambitious pledges in practice (as they should), implementation is incredibly difficult. This is because it requires behavior change, which in turn requires three interrelated factors: motivation, know‐how, and appropriate sociopolitical opportunities for action.^62^ These are often hard to achieve together. Broad policies provide little guidance on how to achieve change in different social, political, economic, and cultural contexts and to gain buy‐in from regional and local communities. As a result, while nations may and do have the ethical responsibility to develop such policies, commitment to international agreements still leaves questions about whose responsibility it is to put such policies into practice and how the goals of such policies can be achieved. In fact, the goals may not be feasible in many countries’ health systems, in which wider financial and social considerations require the balancing of environmental concerns with other values, particularly related to health benefits.^63^ This raises issues of fairness, since meeting carbon targets does not on its own create positive health care outcomes for all.^64^

## Synthesis As We Go

Carbon reduction in health care, and across many sectors, is a moral imperative. As has been shown in other sectors, if health care is to reduce emissions, then change will be required at more than one level. At the same time, what that change could or should look like, how concerns about the deteriorating environment should enter into decision‐making at each of these levels (if at all), and how much priority should be given to each level of change and to particular pathways of decision‐making are still matters of debate. Our analysis has described the ways in which actors at each level are able to respond to their moral obligations and the opportunities and barriers associated with each level.

Other studies on environmental management establish that the levels must not be viewed in isolation from each other or as mutually exclusive. This is crucially important. Transformation has to be created through multiple systems, each of which will intersect with the levels we have mapped out in various ways: First, there must be systemic changes in the governance of health care, production, and consumption, as organized and practiced by societies; this pathway is relevant especially to the international and national levels, as these levels have most control over such governance. Second, structural changes are needed in specific institutions and among constellations of actors. These changes could include introducing policies that aim to reduce environmental harms, aligning workplace norms with resource‐ and energy‐reduction practices, or investing in initiatives and incentives that promote reductions in environmental harms in hospitals and health care systems. Structural changes would occur chiefly at national and regional levels, although they could also occur at local levels through mandatory policy‐making, resource allocation, and cultural change. Third, there is a need for enabling changes that foster human agency, values, and capacities. Such changes can be best envisioned at regional and individual levels^65^ through ethical and pragmatic transformation across health care from research to practice.^66^

Each health care actor ought to endeavor to make changes that lead to carbon reduction—but only insofar as they have the ability to do so. Since individual, regional, national, and international processes for sustainability goals are on different timelines, accepting a sort of “synthesis as we go” approach is wise. Individuals may immediately choose to ask questions about sustainable health care while regional providers take initiatives to make their wards more sustainable, nations deliberate about the best way to implement carbon saving measures, and international bodies include health care emissions in their deliberations. Check‐in points, collaboration, and communication will need to underpin the efforts of all health care actors in their responsibility to be more sustainable, whether that actor is a citizen‐patient, citizen‐clinician, or citizen‐policy‐maker, with particular attention to how much we can ask of actors at some levels when they may not always have the ability to respond.
